# Optogenetic manipulation of inhibitory interneurons can be used to validate a model of spatiotemporal sequence learning

**DOI:** 10.3389/fncom.2023.1198128

**Published:** 2023-06-09

**Authors:** Jackson Rozells, Jeffrey P. Gavornik

**Affiliations:** Center for Systems Neuroscience, Department of Biology, Boston University, Boston, MA, United States

**Keywords:** timing, sequence learning, optogenetics, plasticity, simulation, learned dynamics, experimental validation

## Abstract

The brain uses temporal information to link discrete events into memory structures supporting recognition, prediction, and a wide variety of complex behaviors. It is still an open question how experience-dependent synaptic plasticity creates memories including temporal and ordinal information. Various models have been proposed to explain how this could work, but these are often difficult to validate in a living brain. A recent model developed to explain sequence learning in the visual cortex encodes intervals in recurrent excitatory synapses and uses a learned offset between excitation and inhibition to generate precisely timed “messenger” cells that signal the end of an instance of time. This mechanism suggests that the recall of stored temporal intervals should be particularly sensitive to the activity of inhibitory interneurons that can be easily targeted *in vivo* with standard optogenetic tools. In this work we examined how simulated optogenetic manipulations of inhibitory cells modifies temporal learning and recall based on these mechanisms. We show that disinhibition and excess inhibition during learning or testing cause characteristic errors in recalled timing that could be used to validate the model *in vivo* using either physiological or behavioral measurements.

## 1. Introduction

Mammalian brains are very good at learning to recognize, generate, and predict temporal sequences. This ability is required to plan and execute precisely timed movement sequences, recognize temporal patterns, turn patterns of sounds into words and words into sentences, and is at the root of most complex behaviors. Neuroscience has described neural mechanisms capable of performing this task in specific brain areas, e.g., bird song generation (Long et al., 2010; Egger et al., 2020) or eyelid trace conditioning in the cerebellum (Kalmbach et al., 2010; Siegel et al., 2015), but it is not clear how the mammalian cortex learns explicit representations of either the temporal or ordinal components of sequential information (Bakhurin et al., 2016; Bale et al., 2017; Rabinovich et al., 2022). Multiple studies have shown that visual experience can train the rodent visual system to recognize and represent temporal intervals (Shuler and Bear, 2006; Chubykin et al., 2013; Piasini et al., 2021) and sequences in both passive (Xu et al., 2012; Gavornik and Bear, 2014; Sidorov et al., 2020; Finnie et al., 2021; Price and Gavornik, 2022) and active experimental paradigms (Shuler and Bear, 2006; Fiser et al., 2016). A variety of models have been proposed to explain this cortical ability (reviewed in Keller and Mrsic-Flogel, 2018; Paton and Buonomano, 2018; Price and Gavornik, 2022).

Experiments have implicated cholinergic signaling (Chubykin et al., 2013) at muscarinic receptors (Gavornik and Bear, 2014; Sarkar et al., 2022) as a required component of temporal learning *in vivo*, but it is not known how localized plasticity rules shape circuits to store or access temporal information. One idea is that cholinergic signaling modulates Hebbian-type plasticity to create recurrent excitatory feedback with decay dynamics matching temporal information contained in visual stimulus patterns (Gavornik et al., 2009; He et al., 2015). Experimental work in brain slice (Chubykin et al., 2013; He et al., 2015) supports some aspects of this model, but solid evidence supporting or refuting the basic framework *in vivo* remains elusive. A significant problem is that the activity supporting the temporal information is spread throughout a population of neurons of unknown size and location. V1 plasticity can affect behavior (Cooke et al., 2015; Jurjut et al., 2017; Henschke et al., 2020; Gao et al., 2021) but attempts to tie behavior to specific neural dynamics in context of subtle temporal differences (Levy et al., 2017; Monk et al., 2020; Groen et al., 2022) have been ambiguous and largely defy simple explanations.

Recently, Cone and Shouval proposed a computational model to explain how recurrent timing mechanisms could be used to create sequence representations like those characterized in mouse V1 (Cone and Shouval, 2021). Their model is based around a heterogenous population of neurons that are trained using a biophysically realistic eligibility trace-based learning rule (He et al., 2015; Gerstner et al., 2018). Plasticity of feed-forward projections (Hardy and Buonomano, 2018; Klos et al., 2018; Pereira and Brunel, 2020) from populations of “messenger” neurons convey temporal information from one responsive “column” to the next and are responsible for sequential information cascading from one learned element to the next. While this model is based on cell types and circuit motifs found in the visual cortex and incorporates responses that have been reported in various brain areas (Gavornik and Bear, 2014; Sidorov et al., 2020; Umbach et al., 2020; Reddy et al., 2021), the full set of specific dynamics required for the model to work have not been observed. Even with modern high-throughput methods it would be difficult to directly validate the model *in vivo* for the same reasons discussed above. Since the timing mechanism in the model relies on a synaptically-tuned offset between recurrent excitation and inhibition we reasoned that the model would misreport encoded temporal intervals in a predictable manner if inhibitory activity were artificially perturbed during learning or recall. Accordingly, we modified the neuron model to include simulated optogenetic currents and tested network performance under conditions of increased and decreased total inhibition before and after learning.

Optogenetics can selectively inhibit or excite neurons, providing an approach for the precise control of genetically specified neural populations based on exposure to light (Williams et al., 2013; Mahmoudi et al., 2017; Joshi et al., 2020). There is a large and growing library of opsins that are available for mouse experiments that can be modelled computationally (Nikolic et al., 2013; Williams et al., 2013). Despite this, the literature is largely mute on how optogenetic manipulations can be used as a tool to study temporal representations or validate specific timing models. In this work we created a simulated framework to characterize how spatiotemporal representations produced by the Cone and Shouval model would respond if inhibitory or excitatory opsins were introduced into GABAergic neurons. This approach allows us to describe specific recall failure modes associated with generalized disinhibition or excess inhibition. These failure patterns would be relatively easy to observe *in vivo* with tractable modifications to existing experimental protocols and the approach could be useful to guide experimental probes into the mechanistic underpinnings of spatiotemporal sequence representations in both visual and non-visual cortical areas.

## 2. Materials and methods

### 2.1. Network model

**FIGURE 1:**
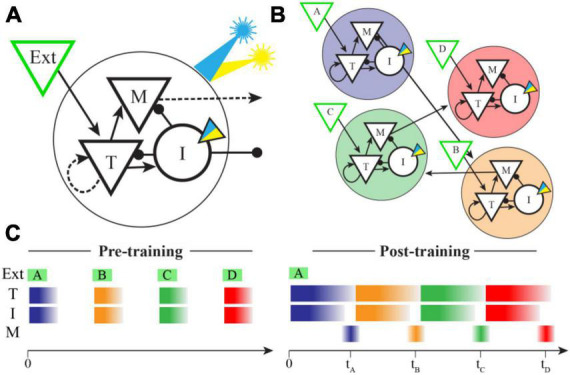
Network model and spatiotemporal representations. **(A)** In the Cone and Shouval model, a functional column contains excitatory (triangles) and inhibitory (circles) neuron populations with connectivity patterns shown. External inputs target both Timer (T) and Inhibitory (I) cells, and both Messenger (M) and I cells send projections to other columns. Dashed arrows represent synapses that are plastic during training. We modified the inhibitory cell model to include an optogenetic current (inset triangle) activated by a simulated laser (blue indicates excitatory, yellow inhibitory). **(B)** By pairing external stimulation of multiple columns in a fixed sequential order during training (shown here as ABCD), the network learns to create ordinal structure via feed-forward M cell projections between columns (for simplicity, lateral inhibitory synapses are not shown). After training, these projections were held constant during optogenetic stimulation trials. **(C)** In naïve networks, external inputs elicit brief bursts of activity during stimulation in both T and I cells, but very little activity in M cells. After training, an external input results in a sustained period of activity in both T and I cells, the duration of which indicates the offset between sequence elements used in training. A brief period of disinhibition when I cells stop firing allows M cells to indicate the end of a temporal interval (times marked) and also convey sequential activity through the network in the absence of additional inputs.

Our network structure is based closely on that published by Cone and Shouval, as is our implementation. Briefly summarizing their model (also see Figure 1), the network is built to have a modular columnar structure with synaptic weights creating distinct populations of “Timer” and “Messenger” cells. Each column consists of both inhibitory and excitatory neurons and receives feed-forward inputs that are uniquely activated by individual elements of the training sequence (e.g., column A receives inputs from sequence element A, column B from element B, etc.). Within each column the excitatory Timer (T) population forms recurrent connections back onto itself and laterally stimulates Inhibitory (I) and Messenger (M) cells. I cells inhibit T and M cells, and M cells send excitatory projections to other columns. The synaptic weight matrix is initialized with small, non-zero values between connected neural populations. Recurrent and intra-columnal excitatory synapses are plastic, and all other populations are assumed to be static.

Neurons are simulated with a standard conductance-based integrate and fire neuron model:


$$C\,\frac{d\,V_{i}}{d\,t}=g_{L}\,\left(E_{L}-V_{i}\right)\,\sigma_{L\,\left(t\right)}+g_{E,i}\,\left(E_{E}-V_{i}\right)+g_{I,i}\,\left(E_{I}-V_{i}\right)+I_{o\,p\,t\,o}$$

where C is the membrane capacitance, *V_i_* is the membrane voltage of neuron i, σ_*L*(*t*)_ introduces random noise into the leak current using a scaled normal distribution, *g_x_* and *E_x_* represent the conductance and reversal potential for a Leak (L), Excitatory (E), and Inhibitory (I) currents, and *I*_*opto*_ is a simulated optogenetic current. Neurons spike when *V_i_* reaches threshold and are held at −61 mV for a 2 ms refractory period.

Synaptic conductance at each synapse *i* is the product of the synaptic weight (set during learning, see below) between two neurons and an activation variable, *s_i_*, that increases by a percent ρ following presynaptic spikes (occurring at times $t_{k}^{i}$) and decays exponentially with a characteristic time constant τ_*s*_:


$$\frac{d\,s_{i}}{d\,t}=-\frac{s_{i}}{\tau_{s}}+\rho \,\left(1-s_{i}\right)\,\sum_{k}\delta \,\left(t-t_{k}^{i}\right)$$

Since the goal of this work is to investigate how temporal reports scale with positive/negative modulation of inhibitory cell activity, we chose to use a simplified optogenetic model that can be adjusted along a single excitation/inhibition axis. Optogenetic current is modeled as:


$$I_{o\,p\,t\,o}=L_{I}\,\left(t\right)\,g_{o\,p\,s\,i\,n}\,\frac{N_{e\,f\,f}}{\sigma_{r}\,\left(t\right)}$$

where *L*_*I*_(*t*) is an indicator function equal to 1 when the simulated laser is active and 0 otherwise and *g*_*opsin*_ sets the strength and valence (inhibitory when *g*_*opsin*_ 0, excitatory when *g*_*opsin*_ >0) of optogenetic activation. The σ_*r*_(*t*) term introduces noise drawn from a scaled standard normal distribution and scales the efficiency term *N*_*eff*_. Different values of *g*_*opsin*_ were used to create inhibitory or excitatory currents in different experiments. This simplified model was motivated by previous works simulating optogenetic currents based on biophysics and light flux (Nikolic et al., 2013; Evans et al., 2016) but is not intended to capture the complex current dynamics through real channelrhodopsin or halorhodopsin proteins. The *N*_*eff*_ parameter was chosen to approximate the average activated state conductance level from Nikolic et al. (2013) and was kept constant throughout all our experiments. Feedforward inputs are modeled by Poisson spike trains that are active during external stimulation and drive excitatory synapses as described above.

Each column in our simulations consists of 400 neurons, and total network size scales linearly with the number of columns. The network was simulated using MATLAB and Euler’s method was used to solve the differential equations. Our simulation code, including all network and model parameters, is available for download at https://gavorniklab.bu.edu/supplemental-materials.html.

### 2.2. Plasticity model and temporal learning

Following Cone and Shouval, we trained the network to represent specific times or sequences using a reinforcement learning rule described previously (He et al., 2015). In this model, summarized here, activity drives dynamic eligibility traces for LTP and LTD in individual synapses between neurons *i* and *j*:


$$\tau^{x}\,\frac{d\,T_{i\,j}^{x}}{d\,t}=-T_{i\,j}^{x}+\eta^{x}\,H_{i\,j}\,\frac{T_{m\,a\,x}^{x}-T_{i\,j}^{x}}{T_{m\,a\,x}^{x}}$$

where *x* indicates either long-term potentiation ($T_{i\,j}^{p})$ or long-term depression ($T_{i\,j}^{d})$. Eligibility traces increase as a function of Hebbian activity across the synapse (*H*_*ij*_, equivalent the simple product of pre- and post-synaptic firing rates) scaled by a learning rate parameter (η*^x^*), saturate at a maximum value ($T_{max}^{x}$) and decay exponentially with a characteristic time constant (τ*^x^*).

During learning, eligibility traces are converted into synaptic weights (*W*_*ij*_) by a reinforcement or reward signal (*R*(*t*)) scaled by a learning rate parameter (η):


$$\frac{d\,W_{i\,j}}{d\,t}=\eta \,R\,\left(t\right)\,\left(T_{i\,j}^{p}-T_{i\,j}^{d}\right)$$

Eligibility traces are set to zero at the beginning of each trial and allowed to develop independently in each plastic synapse based on network activity. The reinforcement signal is modeled as a δ function and non-zero at the time of a putative reinforcement signal elicited by unexpected changes in feed-forward inputs as sequence elements are presented. We trained all networks for 100 trials, which was more than sufficient for $T_{ij}^{p}$ to approximately equal $T_{ij}^{d}$ at the reinforcement time and for both recurrent (T) and feed-forward (M) synaptic weights to stabilize. After the network was trained to represent a specific time or sequence, the plasticity model was turned off and the weight matrix held constant during optogenetic manipulation trials.

### 2.3. Quantifying encoded time

After training, encoded time was determined by challenging the network with a brief pulse of feed-forward input and then determining when activity in the M cells peaked. Since the timing model uses recurrent excitation to encode time, optogenetic stimulation can easily push the network into something like epilepsy due to excess disinhibition for some values of *g*_*opsin*_. Relatedly, excess inhibition can suppress activity to the point that there is no quantifiable temporal report. We used an algorithm to identify and remove “epileptic” trials, defined heuristically as trials where the population average max firing rate of M cells was greater than 180 Hz. If the difference between the maximum and minimum average firing rates of M neurons was less than 5 Hz, the trial was also marked as invalid. When characterizing the timing of sequences, it was also required that the columns activated in the correct order established by the training set. For example, the response of a network trained with ABCD would be marked as invalid if the messenger cells reported ACDB or ABBC. After algorithmic sorting, data was also checked by hand and additional trials were removed if it was visibly obvious that the network was not reporting a well-ordered sequence. Reported time was quantified for valid trials as the time at which the average firing rate of the M cell population peaked.

### 2.4. Statistical analysis

To facilitate robust interpretations, we use violin plots that show an estimate of data distributions (estimated using MATLAB’s ksdensity function) marked to show quartiles and the total data range. All violin plots in a single panel were plotted using the same kernel estimation bandwidth. The statistical effect of optogenetic manipulations during and training and testing was determined using a 2-Way ANOVA test in SPSS.

## 3. Results

### 3.1. The effect of optogenetic manipulation on existing temporal representations

The Cone and Shouval model (Figure 1) represents time via ongoing activity in a population of recurrently connected excitatory neurons trained to decay at a rate matching the timing of disparate environmental cues. Their model also assumes the cortex is divided into functional columns that receive feed-forward inputs representing different external stimuli. Activity in excitatory Timer (T) neurons within a single column is initiated by a brief pulse of feed-forward input. Timer cells drive activity in a population of local Inhibitory (I) neurons and both Timer and Inhibitory neurons form synaptic connections on a population of Messenger (M) neurons. While Timer cells only connect to other cells locally in their own column, the Messenger cells send projections to other circuits (local columns in the original paper, but conceptually other brain areas as well). Plasticity tunes the network so that Inhibitory activity ends before Timer cells, creating a period of disinhibited excitation during which Messenger cells are active. Brief bursts of firing in Messenger cells signals the end of a trained temporal interval. By pairing sequential stimulation of multiple columns at fixed temporal intervals, this model can create synaptic memories that include the relative timing of the external events used to train the network. Since learned “time” is coded via dynamic decay within the Timer cells and conveyed due to precisely timed disinhibition windows, we reasoned that artificial manipulation of the inhibitory population would cause learned times to be misreported in characteristic ways.

**FIGURE 2:**
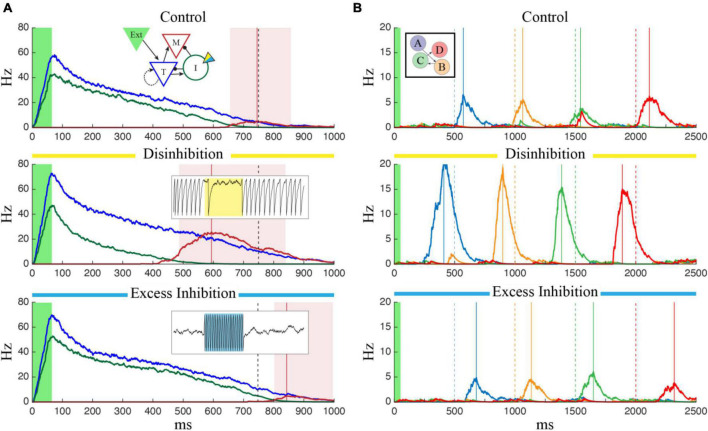
The effects of optogenetic stimulation of temporal representations. **(A)** Each panel shows the average firing rate of T (blue), I (green), and M (red) neuron populations after the network was trained to represent 750 ms (marked by dashed black line). In each case, the green shaded region indicates external input is active, the red vertical line marks the reported “time” corresponding to the peak firing rate of M cells, and the red shaded region indicates that M cells are firing more than one standard deviation above baseline. In the control case (top), there is no optogenetic manipulation and the reported time matches the trained value. The temporal report occurs earlier when the I cells are inhibited (center) and later when they are excited (bottom). In the bottom two plots, optogenetic stimulation was active for the duration of the trial and inset plots show examples of the effect of strong inhibitory (yellow, g_opsin_ = 1.5) or excitatory (blue, g_opsin_ = –1.0) stimulation on single inhibitory neurons (1.5 s of data for each case). **(B)** Optogenetic manipulation of inhibitory cells in a network trained to represent a 4-sequence element shows a similar effect. Compared to the control case (top) disinhibition (middle) and excess inhibition (bottom) cause the network to reactivate faster/slower following brief feed-forward stimulation of column A (green shaded region). Each plot shows the average firing rate of the M cells, color coded by column with the ordinal relationship as shown in the inset. Solid lines indicate report times for each column and dashed lines indicate the target times used during training.

To test this, we modified the neuron model used by Cone and Shouval to include a simulated optogenetically controlled current, *I*_*opto*_. This current was included in the inhibitory population, could be turned on and off at specific timepoints during a network simulation, and could be made to have either an inhibitory or excitatory effect by varying the *g*_*opsin*_ parameter (see Section “2. Materials and methods”). To determine the effect manipulating the inhibitory cells would have on a previously trained temporal interval, we first trained a column to represent a single interval of 750 ms and ran the network over multiple independent trials while activating a simulated laser to perturb activity in the Inhibitory cells. By manipulating the *g*_*opsin*_ parameter, we were able to simulate the effects of both excitatory and inhibitory manipulation of the I cells on temporal reports, quantified as the time of peak firing in the M cells. As shown in Figure 2A, exciting the inhibitory cells (“Excess Inhibition”) increases total inhibition and has the effect of both decreasing the magnitude of M cell firing and, critically, pushing it later in time by diminishing the disinhibitory period responsible for their activity. Inhibiting the inhibitory cells (“Disinhibition”), by contrast, increases M cell firing and causes it to occur earlier. This same effect holds for sequences as well, Figure 2B, with sequence reactivation occurring faster under disinhibition and slower with excess inhibition.

**FIGURE 3:**
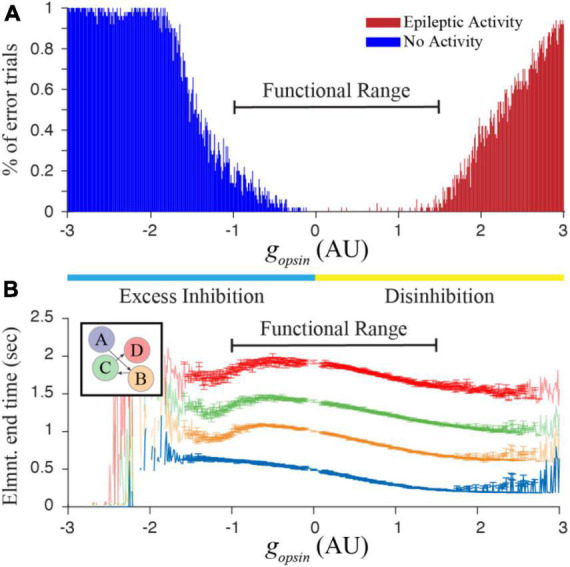
**(A)** Increasing or decreasing optogenetic drive on the I cell population in our simulated network can lead to something resembling epileptic activity when there is too much disinhibition, or a complete lack of activity when there is too much excess inhibition. By calculating the percent of trials showing “errors” that prevent us from quantifying time in a network representing a 4 element sequence (with trained times of 500, 1,000, 1,500, and 2,000 ms), we heuristically defined a functional range for g_opsin_ where at least approximately 80% of independent trials result in interpretable reports of encoded time. **(B)** This functional range produces well-ordered activity across all four columns. Error bars represent the SEM of report times calculated over 1,000 trials conducted for each g_opsin_ value. Large errors, fluctuations, and missing points outside of the functional range result when most trials are removed. Within the functional range, network dynamics (particularly within the first column) generally demonstrate well-behaved timing activity and the majority of trials can be used to characterize the effect of optogenetic manipulations on temporal reports.

The finding from Figure 2 suggests that networks trained to represent different times would respond with characteristic shifts in evoked dynamics following optogenetic manipulation of inhibitory cell activity. To test this more rigorously, we began tracking reported times in trained networks while systematically increasing and decreasing *g*_*opsin*_. It quickly because apparent that there was a limited functional range over which these manipulations produced reasonable results. Too much Excess Inhibition quashes activity in the T and M populations, and too much Disinhibition causes an explosion of activity reminiscent of epilepsy. By algorithmically quantifying these two outcomes, we were able to identify a function range of *g*_*opsin*_ where the network responded to external stimulation with interpretable results (Figure 3) when trained to represent times of in approximately the 0.5–1 s range.

**FIGURE 4:**
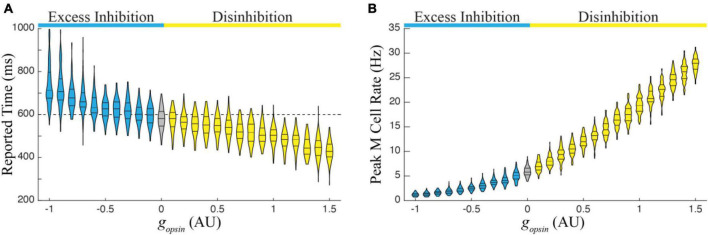
**(A)** The report of an existing temporal interval in a single column trained to a time of 600 ms decreases monotonically with increasing amounts in network disinhibition (yellow) and increases monotonically with increasing levels of excess inhibition (blue) relative to reports when there is no optogenetic manipulation of the I cell populations (gray). **(B)** Relatedly, the peak firing rate of M cells increases with disinhibition and decreases with excess inhibition. Each violin represents times/peaks from 100 trials at the indicated g_opsin_ level and g_opsin_ was varied across the previously defined functional range.

We next ran 1,000 simulations over the entire functional range in a single column trained to represent 600 ms. There were two primary findings from this exercise, both of which follow observations from Figure 2. First, the average duration of reported time increases monotonically with increasing excess inhibition and decreases monotonically with increasing disinhibition (Figure 4A). Second, the peak magnitude of M cell firing during a temporal report decreases with excess inhibition and increases with disinhibition (Figure 4B). Both results represent robust predictions of the model that would be expected *in vivo*.

### 3.2. The effect of optogenetic manipulations during training

The previous section demonstrated that networks which represent time based on the dynamics in Cone and Shouval, with Messenger cells activating during a trained offset between excitatory and inhibitory neurons, will show characteristic misreports of stored time when inhibitory neurons are optogenetically manipulated after training. Since training proceeds based on the co-development and expression of activity dependent LTP and LTD eligibility traces, and stabilizes when the difference between these two is close to zero (see Section “2. Materials and methods”), we reasoned that manipulation of I cells during training would similarly lead to specific predictions about how the network should behave in the presence of post-training manipulations during recall trials. Specifically, we hypothesized that recurrent excitatory weights would increase or decrease in final magnitude to compensate for up or down-regulation of inhibitory activity during training.

**FIGURE 5:**
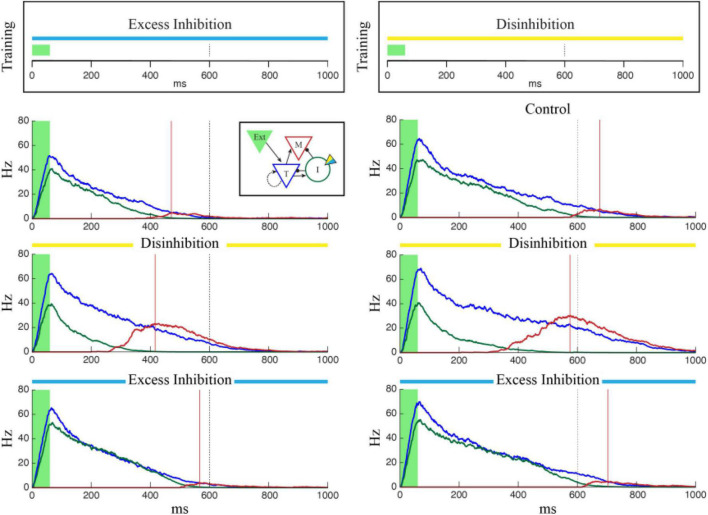
Single column networks were trained (top) to represent 1,000 ms under conditions of Excess Inhibition (**left**, g_opsin_ = –1.0) or Disinhibition (**right**, g_opsin_ = 1.5) after which the networks were tested under Control conditions (no opto stimulation), Excess Inhibition (blue), or Disinhibition (yellow). In both cases, the network reported the correct time under conditions in which it was trained. When conditions did not match training, the Excess Inhibition network under reported the encoded time and the Disinhibition network over reported it. Plots show representative average population activity levels under specified conditions after training, color coded as in the inset. The dashed black line is the training target and the red lines are encoded time values reported by the M cells.

To test this, we modified our training procedure to include constant stimulation of either excitatory or inhibitory opsins in I cells during training (Figure 5, top). In each case, a single column was trained to represent 600 ms with constant optogenetic stimulation during each training trial. A control network was trained to the same time without any manipulation. Learning dynamics in all three cases were equivalent, with the networks reaching learning saturation after approximately the same number of trials and no obvious effect of stimulation on learning rate. After training, however, the time reported by the network depended on the conditions experienced during training. Networks trained with excess inhibition reported the correct time when I cells were excited but under-reported the trained time when the network was disinhibited or no optogenetic manipulations occurred (Figure 5, left). Similarly, networks that were disinhibited during training accurately reported the training time when there were again disinhibited. When subjected to excess inhibition or control conditions, they tended to over-report the encoded time (Figure 5, right).

**FIGURE 6:**
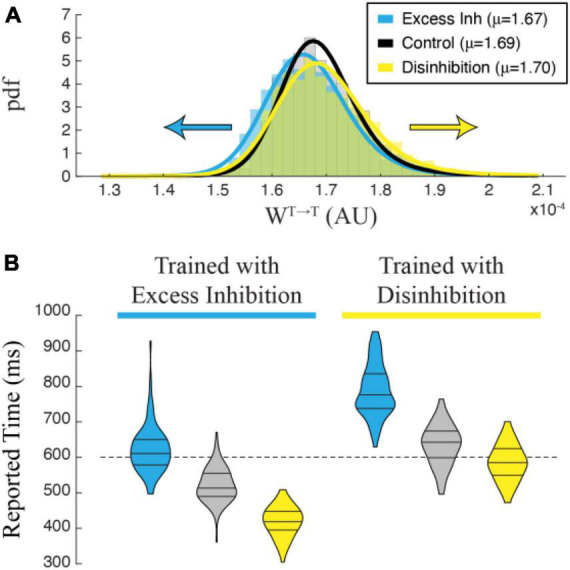
**(A)** Recurrent excitatory weights trend larger in a network trained with Disinhibition (yellow) than in a Control (black) network trained without optogenetic stimulation, while weights trend smaller in a network trained with Excess Inhibition (blue). The graph shows an estimated pdf of the weight distribution color-coded for each case, with solid lines showing pdfs of Stable distributions fit to each data set. Average values for each set of weights are reported in the legend. **(B)** Violin plots showing the distribution of time reports after 100 trials in each condition. Post-training trials that match the training condition are the most accurate, while trials in differing conditions skew negative (Excess Inhibition training) or positive (Disinhibition training). A 2-way ANOVA shows a significant interaction between conditions during training and testing (*F*_2,594_ = 13.905, *p* < 0.001) with significant main effects of training (*F*_1,594_ = 1007.960, *p* < 0.001) and testing (*F*_2,594_ = 646.612, *p* < 0.001). All *post-hoc* comparisons were highly significant with Bonferroni-corrected *p*-values < 0.001.

To understand why manipulating inhibitory cells during training has this effect on reported time, we plotted the distribution of recurrent excitatory synaptic weights within the T population. A representative example in Figure 6A shows that networks trained with disinhibition tend toward larger recurrent excitatory weight matrices than control networks trained without manipulation. Recurrent weights in networks trained with excess inhibition similarly trend smaller. This finding matched our intuition that training would proceed until the activity-level offset between potentiating and depressing eligibility traces was equal to zero regardless of external manipulations, meaning that recurrent excitatory weights had to increase or decrease to compensate for representational shifts caused by our artificial up or down regulation of inhibitory activity. When cued to report their encoded times without any stimulation, trained networks exhibit evoked dynamics reflecting the shifted weight matrices with stronger weights resulting in slower decay times and weaker weights in faster decay times. Correct temporal dynamics can be reestablished by testing the network with the same manipulation used during training, and the misreport is exaggerated when the valence of optogenetic stimulation is reversed. As shown in Figure 6B, these effects are robust and differentiate between cases at the population levels. As with the effects of optogenetic stimulation on a previously trained network, these findings can be considered a strong prediction of how temporal representations based on the Cone and Shouval model would be expected to behave when inhibitory cells are optogenetically manipulated *in vivo* during temporal learning trials.

## 4. Discussion

The concept of “time” is notoriously difficult to pin down, so perhaps it’s not surprising that we don’t have a good handle on the neurobiological basis of temporal perception. Despite a good deal of interest in the subject, there are still many more questions than answers about how time is represented in the brain. Even less is known about how activity dependent plasticity shapes cortical circuits to create memories that include temporal information, or how the brain uses this stored information to predict how events will unfold in time. One of the reasons for this is that we haven’t identified many neural correlates of learned time that can be easily measured and linked to either physiological activity or behavioral outcomes. A variety of models have been developed to account for temporal perception, but often at a level of abstraction that makes it difficult to tie back to specific biology.

The visual system, including primary cortical areas, has emerged over the last decade as an unexpectedly rich system to study time, sequence learning, and temporal predictions. Starting with the demonstration that cells in rat V1 can learn evoked dynamics that predict when a reward will be delivered following visual stimulation (Shuler and Bear, 2006), visual cortex has also been shown capable of learning to produce trajectories (Xu et al., 2012; Ekman et al., 2017), recognizing sequences (Gavornik and Bear, 2014; Sidorov et al., 2020; Ekman et al., 2023) and making predictions about expected visual inputs (Fiser et al., 2016; Leinweber et al., 2017; Pakan et al., 2018; Homann et al., 2022) in various animals including humans. The Cone and Shouval model was developed to explain how a recurrent timing mechanism developed to explain interval timing in V1 (Gavornik et al., 2009; Gavornik and Shouval, 2010) could be extended to explain spatiotemporal sequence learning. While the model makes specific predictions about the types of cells you would expect to see in a brain region using this mechanism (T, I and M cells), the presence of cells with these response types would not validate the model’s core mechanism since it was specifically designed to produce these cell types based on previous observations (Huertas et al., 2015).

In this paper we show that a unique aspect of this model’s timing mechanism presents a viable target for experimental validation. Our inclusion of a simulated optogenetic current in the membrane voltage equation of inhibitory cells in a simulated network demonstrates that excess inhibition tends to lengthen the duration of temporal reports in trained networks and disinhibition tends to shorten them. We also show that a network trained with either excess inhibition or disinhibition will recall incorrect times, underestimating and overestimating, respectively. The implication is that optogenetic stimulation targeting inhibitory interneurons can be used validate the model by artificially slowing down or speeding up learned internal clocks. Put another way, if an animal learns to represent a specific temporal profile using the mechanisms proposed by Cone and Shouval, optogenetic excitation of inhibitory neurons participating in the representation should cause the animal’s internal clock representing the time to run slow. Optogenetic silencing of the inhibitory neurons should cause the clock to run fast. Any physiology or behavior dependent on the temporal representation should similarly shift and the same approach would be valid whether the coding occurs in V1 or some other cortical region.

We targeted inhibitory neurons because of our analysis suggested an important role in producing organized readouts of stored intervals, but there are additional reasons to focus on them as well. As a practical matter, there are a variety of tools that can be used to manipulate inhibitory activity. The literature is full of examples showing how the Cre-LoxP system (Kim et al., 2018) can be used to control conditional expression of both excitatory and inhibitory opsins in GABAergic cells generally or within specific subpopulations of PV or SOM, or VIP positive neurons. While the Cone and Shouval model does not differentiate between these classes of inhibitory cells, it does include distinct subsets of inhibitory neurons associated with the T and M populations (these are included in our simulations). We intentionally chose to treat all inhibitory cells as equivalent so that our results would be more relevant to understanding how temporal representations would behave if optogenetic proteins were targeted to inhibitory cells broadly using something like the GAD promoter (Taniguchi, 2014). This model does not address how temporal representations would be expected to change if expression were restricted to sub-populations. Based on cortical circuit motifs, however, it seems likely that dendrite targeting SOM cells are a likely candidate for involvement in this model. The same conditional expression strategies could also be used to deliver inhibitory or excitatory versions of DREADDs (Smith et al., 2016), which could be an effective tool to maintain elevated or diminished inhibitory activity over extended training sessions where phototoxicity might be a concern. We also considered whether optogenetic manipulation of excitatory cells would be useful to validate this model but decided this was not a useful approach after pilot work revealed that excitation and inhibition of excitatory populations leads to disordered representations with columns activating randomly regardless of training.

We used a standard ANOVA to show that different levels of optogenetic manipulation during training and testing result in statistically different temporal reports in our simulated networks. While this is useful to demonstrate that the effect size of these manipulations is large enough to expect similar experiments *in vivo* would produce statistically significant outcomes, we do not think that these statistics are necessary to convincingly link the overall trends to specific elements of the network timing structure. Because the precise statistics depend entirely on the parameters used for a particular run, these numbers have little predictive power when making experimental design decisions *in vivo*. This is particularly true because we chose to use a simplified model of optogenetic current rather than more biophysically realistic alternatives (e.g., knowing that report times in our simulated network achieve statistical significance only when the g_opsin_ parameter changes by some number of arbitrary units cannot be extrapolated to answer experimentally relevant questions like “what is the expected effect size if I use 10 mW of laser power?”). While a more realistic optogenetic model could, in principle, provide more guidance for designing experiments based on this work we do not think this possibility is worth the additional complexity since many of the relevant parameters are difficult to either estimate or control *in vivo* (e.g., cell membrane area or protein expression levels) and since the many parameters in the Cone and Shouval model itself are rough approximations.

This work does not address how other timing models would respond to similar manipulations of inhibitory cells. While there are compelling reasons to think that the plasticity responsible for learning temporal representations occur in local cortical circuits (e.g., learning can be prevented through localized infusions of plasticity blocking drugs), it is possible that learning requires timing mechanisms outside of the cortex. It was recently shown, for example, that hippocampal lesions can prevent sequence representations from forming in the primary visual cortex (Finnie et al., 2021) and place-cell like spatial navigational information is available in V1 (Saleem et al., 2018). This might suggest that temporal representations in V1 require access to hippocampal time cells (Eichenbaum, 2014). In this case, it is unlikely that manipulating inhibitory cells in the cortex would change the duration of coded time. A variety of works have considered how neuromodulation effects working memory models based on attractor dynamics in recurrently connected networks similar to ours (Brunel and Wang, 2001; Wang et al., 2004; Chumbley et al., 2008) but tend to focus on analyzing the extent to which the memory can be maintained in the presence of distractors rather than considering how a timing circuit based on these mechanisms would be effected by inhibitory disruptions. It is possible that optogenetic manipulation of inhibitory cells would similarly affect temporal readouts in other timing models, potentially making it difficult for experimental work to uniquely validate the Cone and Shouval model. Further work would be needed to understand how other specific timing models depend on inhibitory dynamics.

The main takeaways from this work are that trained temporal reports in the Cone and Shouval model are particularly sensitive to lateral inhibition, and that optogenetic manipulation of inhibitory cells *in vivo* should slow down or speed up recalled temporal memories in predictable ways. This is true whether manipulations occur after specific timing has already been learned or during training. While further refinements of the model would allow more precise predictions, these results are sufficient to motivate and guide future experiments testing whether the brain uses an offset between decaying excitatory and inhibitory activity to signal time in the cortex.

## Data availability statement

The original contributions presented in this study are included in the article/supplementary material, further inquiries can be directed to the corresponding author.

## Author contributions

JR and JG: conception and experimental design, data analysis, visualization, and writing the manuscript. JR: coding. Both authors contributed to the article and approved the submitted version.
